# Relationships between Potentially Toxic Elements in intertidal sediments and their bioaccumulation by benthic invertebrates

**DOI:** 10.1371/journal.pone.0216767

**Published:** 2019-09-19

**Authors:** Tom Sizmur, Lily Campbell, Karina Dracott, Megan Jones, Nelson J. O’Driscoll, Travis Gerwing

**Affiliations:** 1 Department of Geography and Environmental Science, University of Reading, Reading, England, United Kingdom; 2 Department of Biology, University of Victoria, Victoria, British Columbia, Canada; 3 North Coast Cetacean Research Initiative, Ocean Wise Conservation Association, Prince Rupert, British Columbia, Canada; 4 Department of Earth & Environmental Sciences, Acadia University, Wolfville, Nova Scotia, Canada; 5 Ecosystem Science and Management Program, University of Northern British Columbia, Prince George, British Columbia, Canada; University of Waikato, NEW ZEALAND

## Abstract

The bioaccumulation of Potentially Toxic Elements (PTEs) by benthic invertebrates in estuarine sediments is poorly understood. We sampled and analysed PTEs in sediments and benthic invertebrates from five sites in the Skeena Estuary (British Columbia, Canada), including sites adjacent to an abandoned cannery and a decommissioned papermill. Our aim was to elucidate baseline levels of PTE concentrations at sites that may be recovering from disturbance associated with prior industrial development and identify organisms that could be used to biomonitor the impact of future industrial developments. There was no indication that sediments of the salmon cannery were polluted, but acidic sediments adjacent to the papermill contained elevated concentrations of Cd, Cr, Hg and Pb. Benthic invertebrate community assemblages confirm that sediments have mostly recovered from prior industrial development associated with discharge of papermill sludge. Overall, we did not observe any relationship between PTE concentrations in the sediment and PTE concentrations in invertebrate tissues. However, we did observe a negative relationship between sediment pH and the Biota-Sediment Accumulation Factor (BSAF) of most PTEs for Oregon pill bugs (*Gnorimosphaeroma oregonensis*). *G*. *oregonensis*, observed at all sites, feeds on the fibers associated with the papermill discharge. Thus, *G*. *oregonensis* is a useful biomonitors for quantifying the impact of the decommissioned papermill, and are candidate biomonitors for assessing the impact of similar industrial development projects on intertidal ecosystems.

## Introduction

Intertidal estuarine habitat is both ecologically and economically important, providing nursery habitats to several species, such as juvenile pacific salmon, that support important commercial fisheries [1]. Estuaries are often very biologically diverse, exhibiting an abundant array of flora and fauna [2–6]. One abundant taxonomic group often found in estuaries are benthic invertebrates [5, 7]. Benthic invertebrates are often used as indicators of environmental pollution since they live in sediments and are directly exposed to any contaminants located within the sediment. Further, they are prey for many commercially important fish species, often responding to pollution before commercially viable species are negatively impacted, warning of the potential threat of pollution [8–10]. Benthic invertebrates inhabiting estuaries are inherently resistant to physical and chemical change as they have adapted to living in a dynamic environment with wide spatial and temporal ranges of chemical and physical properties, such as pH, redox potential, salinity, and particle size [11–13].

Potentially Toxic Elements (PTEs) are naturally occurring elements (e.g. Arsenic Cadmium, Cobalt, Chromium, Nickel, Mercury, Lead and Zinc) found in all ecosystems, but are typically associated with contamination and potentially toxic to plants, animals and humans when present in elevated concentrations [14]. Anthropogenic activities have impacted the biogeochemical cycling of Potentially Toxic Elements (PTEs) in even the most remote ecosystems on our planet [15, 16]. Because the chemical and physical properties of estuarine sediment influence the bioavailability of PTEs to benthic invertebrates and are temporally and spatially dynamic, it is difficult to predict the impact of industrial development on the fate and impact of PTEs [17]. As such, we do not currently understand the relationship between sediment properties, food web structure and PTE bioaccumulation by sediment-dwelling invertebrates well enough to predict the impact of pollution from industrial developments upon the estuarine environment. Quantifying the disruption of natural biogeochemical cycles of PTEs by anthropogenic activities is difficult if concentrations prior to industrial development are unknown. Therefore, it is important to conduct baseline surveys to assess the bioavailability and bioaccumulation of PTEs by organisms to predict the likely impact of further pollution on the ecosystem.

The Skeena is British Columbia’s second largest river and the estuary provides important nursery habitats for juvenile salmon [3, 18]. Coastal areas to the north of the estuary surrounding the small port cities of Prince Rupert and Port Edward have been extensively developed [19–22]. Industrial developments included an international port, a papermill, and several canneries. Findings of previous surveys of the benthic invertebrates inhabiting the intertidal sediment in the Skeena estuary reveal an infaunal community that is relatively undisturbed at the estuary scale, but which still shows the scars of historic industrial developments at specific locations [5, 11, 23, 24]. High densities of amphipods throughout the Skeena estuary [5, 11] suggests that current disturbances to intertidal areas are relatively limited. Similarly, 40 intertidal invertebrate species are observed in this area, including multiple species at all trophic levels within the food web [5, 11], and such a complex community is often associated with non-disturbed habitats [21, 25–27]. Conversely, disturbed sites are often more easily invaded by invasive species [28, 29], and Capitellidae (*Capitella capitata* species complex) polychaetes are often observed in disturbed habitats, particularly areas that have been organically enriched [21, 25, 30, 31]. Capitellidae polychaetas have a clumped distribution within the Skeena estuary and can be locally abundant at the scale of a 1m^2^ plot [5, 11]. Similarly, abundant but localised populations of the invasive cumacean, *Nippoleucon hinumensis*, have also been observed in the Skeena estuary [5, 11], and on other historically impacted mudflats along BC’s north coast [31]. While the universal presence and high abundances of amphipods, coupled with the complexity of the intertidal community at the estuary scale strongly suggest that these intertidal communities are relatively undisturbed, the locally abundant populations of invasive species and Capitellidae polychaetes offer contradictory evidence. These indicators of disturbance suggest that either localised disturbances are currently occurring, or this biological signal is a remnant of past industrial developments. To the best of our knowledge, no current industrial developments are occurring in the Skeena estuary that can explain the localised indicators of disturbance observed in the biological community [5, 11, 24, 31]. Therefore, we hypothesise that localised disturbance in the Skeena estuary is a legacy of past industrial developments [5, 11]. It is likely that these disturbances are at least partially related to discharge from the papermill on Watson Island, which was released into the immediate near shore area (Porpoise Bay), strongly depressing the invertebrate communities in this area during the 1970s [20, 32, 33]. As such, we postulate that these intertidal areas have been passively recovering from this disturbance for ~50 years. However, it is not clear the extent to which the papermill discharge has been buried by fresh ‘clean’ sediment and is no longer bioavailable to benthic invertebrates.

Considerable developments have been proposed in the Skeena estuary, including oil and gas pipelines, super-tanker routes, potash loading facilities, and a liquid natural gas (LNG) terminal. The Skeena salmon run contributes an estimated $110 million dollars annually to the local economy [34], so pollution of the intertidal nursery habitat of the Skeena Estuary could have devastating consequences on both the economy and ecosystem [35, 36]. It is therefore critical to predict the impact that future developments will have on the bioaccumulation of PTEs by benthic invertebrates inhabiting intertidal sediments of the Skeena Estuary. Our aim was to investigate the extent to which intertidal sediments in the Skeena Estuary are contaminated by historic industrial developments and identify organisms that could be used to biomonitor the impact of future industrial developments. As such, the objectives of this study were to (i) explore the relationship between sediment depth, sediment properties and the fate of PTEs from historic industrial development in the Skeena Estuary, and (ii) quantify the impact of PTEs on the community assemblages of benthic invertebrates inhabiting intertidal sediments and the bioaccumulation of PTEs into their tissues. To achieve these objectives we conducted a survey of sediments and invertebrates at five intertidal sites north of the Skeena river mouth to examine baseline levels of PTE concentrations in sediments and benthic invertebrates at sites that may be recovering from previous disturbances and subject to future industrial development.

## Methods

### Field sampling

**Fig 1:**
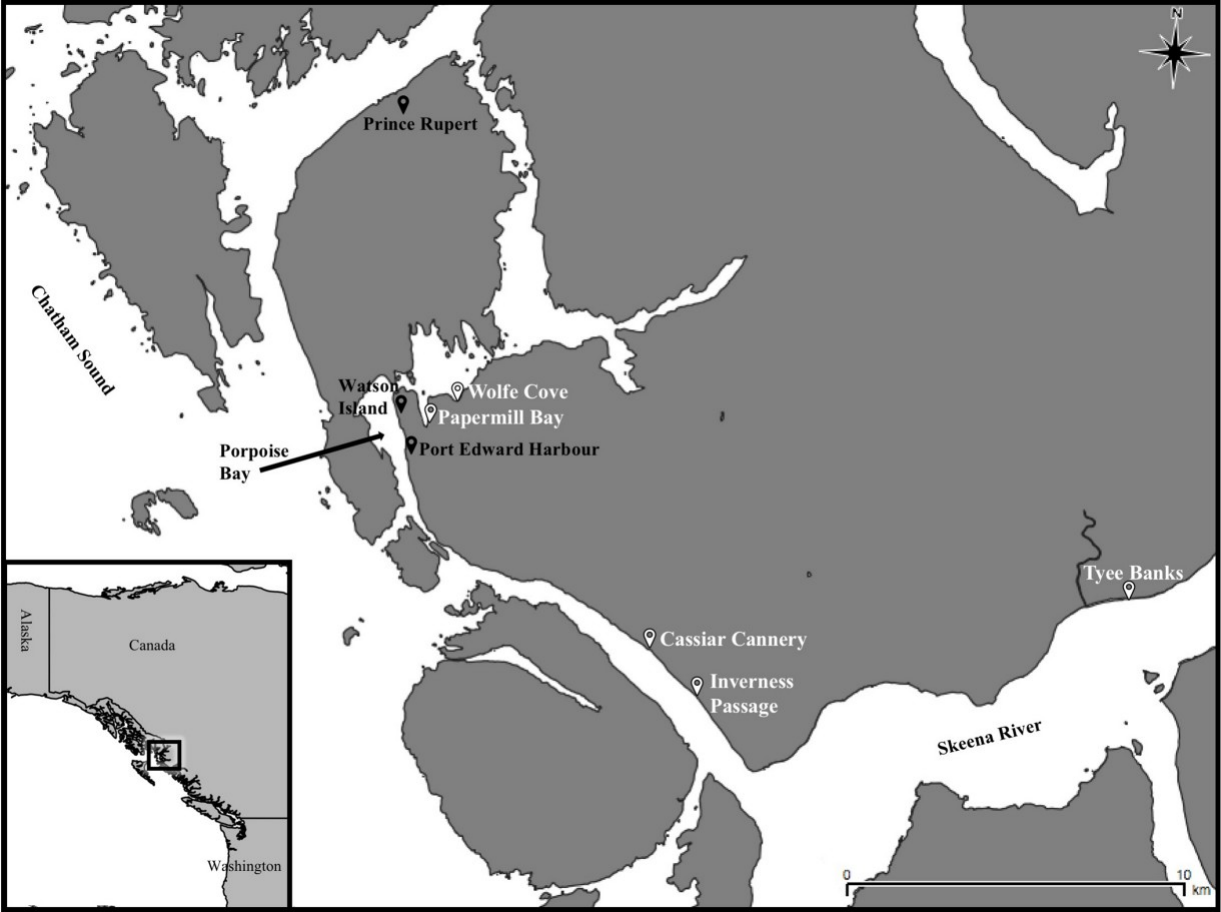
Map showing sites adjacent to the Skeena Estuary, British Columbia, Canada where intertidal mudflats at Papermill Bay, Wolfe Cove, Cassiar Cannery, Inverness Passage, and Tyee Banks were sampled during this project.

Sediment cores and benthic invertebrates were collected from five intertidal sites north of the Skeena river mouth (Fig 1) during July 2017: Cassiar Cannery (CC), Inverness Passage (IP), Papermill Bay (PB), Wolfe Cove (WC), and Tyee Banks (TB). Cassiar Cannery (N54° 10’ 40.4, W130° 10’ 40.4) is a former salmon cannery that closed in 1983 and is now an ecotourism lodge. Inverness passage (N54° 10’ 05.9, W130° 09’ 40.4), a mudflat ~3 km closer to the Skeena mouth than Cassiar Cannery, has intertidal habitat similar to Cassiar Cannery, but was never directly impacted by a cannery or other anthropogenic development. Inverness passage was thus selected as a reference site proximal to Cassiar Cannery. Papermill Bay (N54° 13’ 59.3, W130° 17’ 07.5) is a small bay located directly adjacent to a large decommissioned papermill. The papermill was closed in 2001, ceasing all operations and discharge [37–39]. Wolfe Cove (N54° 14’ 33.0, W130° 17’ 34.5) is a mudflat located approximately 1 km from the papermill, selected as a reference site proximal to Papermill Bay. Finally, Tyee Banks (N54° 11’ 59.1, W129° 57’ 36.7) is located 20km upstream from the mouth of the Skeena River on a large intertidal mudflat. At some point in the past, this area had a small-scale sawmill operating and accumulations of sawdust and woodchips are still present in the upper intertidal sediment. Tyee Banks was selected as a reference site in a remote location, much further away from Cassiar Cannery and Papermill Bay than the proximal reference sites. As this work was carried out on nearshore habitat in Canada, focusing upon common intertidal invertebrates, no permits or specific permissions were required to conduct this work.

Sediments were sampled by pushing polycarbonate cores (5 cm diameter, 20 cm length) into the mud with a rubber mallet, digging out with a spade, and wrapping with cling film, before extruding and dividing into strata; 0–5 cm, 5–10 cm, 10–15 cm and 15-20cm. Sediment samples from each strata were dried at 40°C for 48 hours and shipped to the UK for analysis. A total of 15 cores were collected from each site along five transects comprising three samples taken from the upper, mid and lower shore in a random stratified sampling design [40]. Sediments from the upper, mid and lower shore (but the same strata) were pooled and homogenized prior to analysis. At each location that a sediment core was collected, two more cores (7 cm diameter) were taken to a depth of at least 5 cm (well into the anoxic layer [41]), transferred to a plastic bag in the field and then passed through a 250μm stainless steel sieve to retain sediment-dwelling benthic infauna (animals living in the sediment). This infauna sampling strategy was supplemented by opportunistic digging in areas of the site in which infauna were clearly present (e.g. burrow openings or surface casting) to collect macrofauna (animals retained on 1mm mesh). All invertebrates found in sufficient quantities for chemical analysis (0.5 g dry weight) were identified to the species level [5, 11], pooled into a single sample for each site, rinsed in deionised water, frozen, shipped to the UK, and freeze-dried prior to analysis.

### Laboratory analysis

The particle size distribution of sediments was determined using laser granulometry (Malvern Mastersizer 3000). Sub-samples were then ground to a fine powder using an agate ball mill and analysed for total organic carbon and nitrogen content using a Thermo Scientific Flash 2000 Organic Elemental Analyser. Sediment pH was determined in a soil-water suspension after shaking with water for 15 min at a 1:10 w/v ratio based on BS7755-3.2 [42]. The total concentration of PTEs in sediments was determined by ICP-MS (Inductively Coupled Plasma Mass Spectrometry) analysis of 0.5 g sediment samples digested in reverse aqua regia (9 ml of nitric acid and 3 ml of hydrochloric acid) using a MARS 6 microwave digestion system, based on EPA [43]. After preliminary analysis of a large range of PTE concentrations, the elements selected for further investigation were Cadmium (Cd), Cobalt (Co), Chromium (Cr), Nickel (Ni), Lead (Pb) and Zinc (Zn). The total concentration of Mercury (Hg) in sediments was determined using thermal degradation–gold amalgamation atomic absorbance spectroscopy as outlined in EPA [44] using a Nippon MA-3000 analyser. The bioavailability of PTEs to biota was estimated by extracting 2.5 g of sediments with 25 ml of 0.05M EDTA (Ethylenediaminetetraacetic acid) at 20°C for one hour, centrifuging, filtering and analysing PTEs (As, Cd, Co, Cr, Cu, Ni, Pb and Zn) in the extract using ICP-OES (Inductively Coupled Plasma Optical Emission Spectrometry). The total concentrations of PTEs in invertebrates were determined by ICP-MS analysis of 0.5 g of sample digested in slightly diluted nitric acid (2 ml of ultra-pure water and 8 ml of nitric acid) using a MARS 6 microwave digestion system. Details of quality control undertaken during laboratory analysis can be found in the supporting information file.

### Statistical analysis

A Geoaccumulation index was calculated following [45] to determine if the potentially polluted sites (Cassiar Cannery and Papermill Bay) are polluted with respect to proximal (Inverness Passage and Wolfe Cove, respectively) and remote (Tyee Banks) reference sites using the following equation:

Igeo=Log2Cn1.5×Bn

when *C_n_* = Concentration measured in sediment at potentially polluted site and *B_n_* = Concentration measured in sediment at reference site.

Sites were classified as unpolluted if Igeo ≤ 0, unpolluted to moderately polluted if 0 ≤ Igeo ≤ 1, moderately polluted if 1 ≤ Igeo ≤ 2, moderate to strongly polluted if 2 ≤ Igeo ≤ 3, strongly polluted if 3 ≤ Igeo ≤ 4, strongly to extremely polluted if 4 ≤ Igeo ≤ 5, and extremely polluted if Igeo ≥ 5.

Biota-Sediment Accumulation Factors (BSAFs) were calculated by dividing the concentrations of PTEs in the invertebrate tissues by the average concentration in the sediments from the site from which the invertebrates were collected (average across all transects and depths).

The influence of site and sediment depth on sediment PTE concentrations and properties was quantified using analysis of variance (ANOVA) and permutational multivariate analysis of covariance (PERMANCOVA) [46, 47]. The relationship between PTE concentrations and sediment properties was first tested using PRIMER’s RELATE function [48]. This function compares two resemblance matrices looking for any relationships. Relationships were further explored using principal component analyses (PCA) on the variance-covariance matrix of all sediment PTE and sediment property data. Relationships between the PTE concentrations observed in the sediments and in the collected invertebrates were examined in several configurations using RELATE and plotted using non-metric multidimensional (nMDS) scaling plots. A full description of the statistical analysis undertaken is provided in the supporting information file and outputs provided in S1, S2 and S3 Tables.

## Results and Discussion

### Inverness Passage, Wolfe Cove and Tyee Banks are suitable reference sites

**Fig 2:**
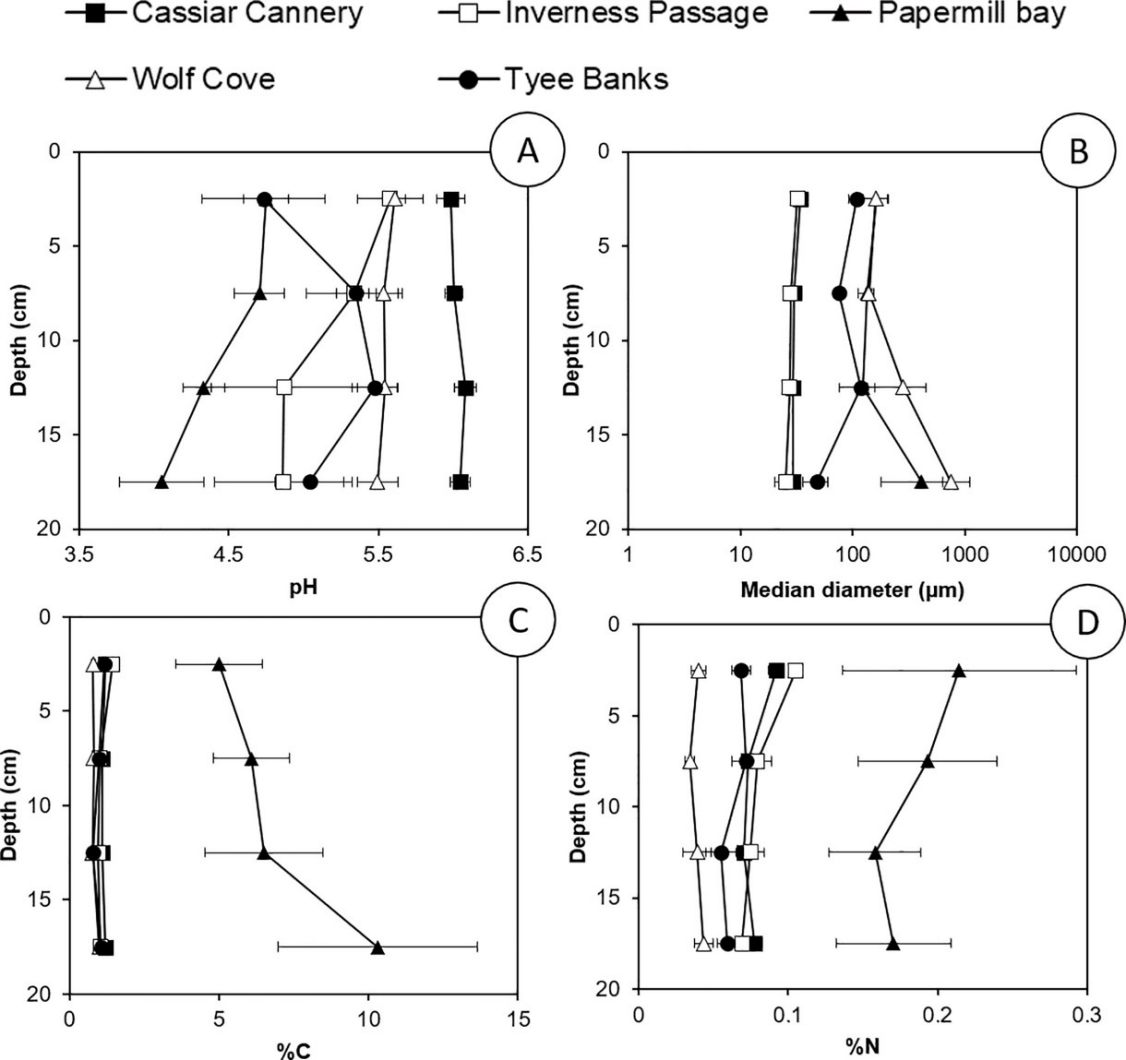
Sediment properties; Sediment pH (A); Median sediment particle diameter (B); Percentage Organic Carbon content (C); and Percentage Total Nitrogen content (D) presented at four depths in sediments sampled from Cassiar Cannery, Inverness Passage, Papermill Bay, Wolfe Cove and Tyee Banks intertidal mudflats.

The median particle diameter of sediments was used to determine whether sediment deposited at different sites were similar to each other so that the suitability of reference sites could be assessed. The PERMANCOVA analysis indicates that properties of sediments (pH, median particle diameter, C and N) are significantly influenced by both site and sediment depth, with site explaining more than 50% of the observed variance in the data (S1 Table). There was no significant (p > 0.05) difference in the median sediment particle diameter between Inverness Passage and Cassiar Cannery, or between Wolfe Cove and Papermill Bay (Fig 2 and S3 Table). This observation supports our assumption that the sediment deposited in the sites potentially contaminated by industrial development (Cassiar Cannery and Papermill Bay) and their respective proximal reference sites (Inverness Passage and Wolfe Cove) have the same geogenic origin and are subject to a similar depositional environment. Thus, any differences in sediment chemical properties between potentially contaminated sites and their proximal reference sites we infer is due to anthropogenic influences.

Tyee Banks had a significantly (p < 0.05) larger median particle size than Inverness Passage and Cassiar Cannery and a significantly (p < 0.05) smaller median particle size than Wolfe Cove and Papermill Bay (Fig 2 and S3 Table). The location of Tyee Banks is closer to the mouth of the Skeena River and, therefore, only receives sediment from the Skeena, whereas the other four sites may also receive sediment from the Nass River, to the north of the Skeena Estuary [49]. However, because Tyee Banks is much further from potential pollution sources, the site can be used to determine whether proximal reference (Inverness Passage and Wolfe Cove) sites are contaminated with PTEs from the adjacent historic industrial developments.

### Papermill Bay sediments are contaminated with Cd, Cr, Hg and Pb

**Fig 3:**
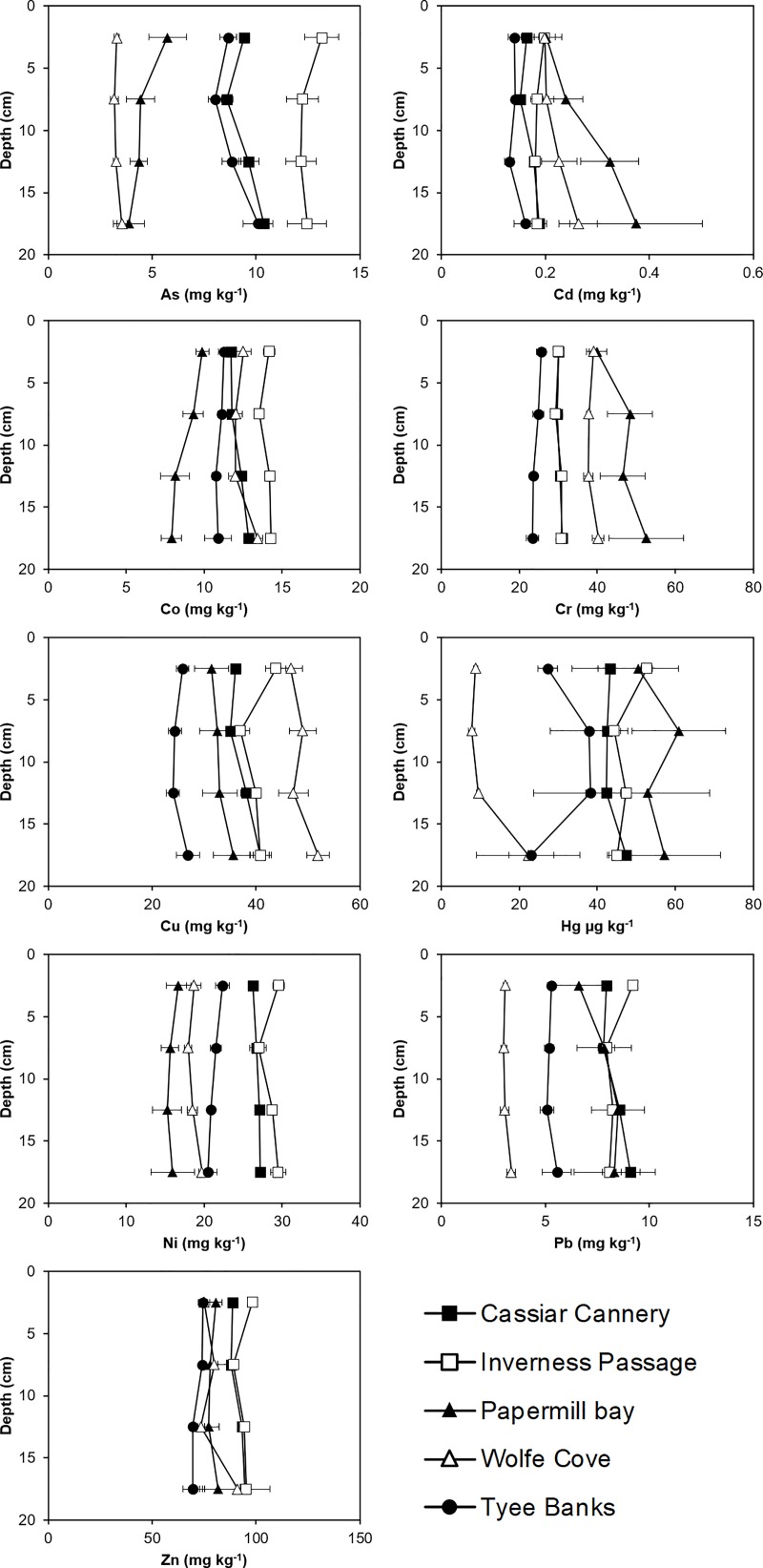
Total concentrations of Potentially Toxic Elements (As, Cd, Co, Cr, Cu, Hg, Ni, Pb and Zn) with depth in sediments sampled from Cassiar Cannery, Inverness Passage, Papermill Bay, Wolfe Cove and Tyee Banks intertidal mudflats.

Concentrations of PTEs at potentially polluted sites were compared with reference sites to assess whether the potentially polluted sites are contaminated. The PERMANCOVA analysis (S2 Table) indicated significant differences in sediment total and EDTA extractable PTE concentrations between different sites, but not between different depths. We observed significantly (p < 0.05) greater concentrations of As, Cr, Hg and Pb in the Papermill Bay sediments than the proximal reference sediments at Wolfe Cove (Fig 3 and S3 Table). However, Co, Cu and Ni concentrations were significantly (p < 0.05) lower in the Papermill Bay sediments (Fig 3 and S3 Table). The Geoaccumulation index, using Wolfe Cove as an uncontaminated reference site, indicated that Papermill Bay is ‘unpolluted’ with As, Cd, Co, Cr, Cu, Ni and Zn, but ‘unpolluted to moderately polluted’ with Pb and ‘moderately polluted’ with Hg (Table 1). When Tyee Banks was used as the reference site, Papermill Bay is classified as ‘unpolluted to moderately polluted’ with Cd, Cr and Hg. Thus, there is evidence to suggest that the sediments in Papermill Bay are contaminated with Cd, Cr, Hg and Pb, most likely emanating from the sludge [50] discharged by the decommissioned papermill on Watson Island (Fig 1).

**Table 1:** Geoaccumulation index (Igeo)^a^ for potentially toxic elements in sediments from Cassiar Cannery (CC) and Papermill Bay (PB) using reference sites Tyee Banks (TB), Wolfe Cove (WC) and Inverness Passage (IP).

Site	Reference site	As	Cd	Co	Cr	Cu	Hg	Ni	Pb	Zn
CC	IP	-0.99	-0.71	-0.78	-0.57	-0.69	-0.69	-0.67	-0.58	-0.63
	TB	-0.48	-0.33	-0.44	-0.27	-0.01	-0.13	-0.26	0.08	-0.24
PB	WC	-0.10	-0.25	-1.08	-0.32	-1.14	1.61	-0.82	0.74	-0.60
	TB	-1.51	0.39	-0.90	0.35	-0.19	0.20	-1.01	-0.02	-0.45

^a^ Geoaccumulation index is calculated as Igeo=Log2Cn1.5×Bn when *C_n_* = Concentration measured in sediment at site and *B_n_* = Concentration measured in sediment at reference (background) site.

Sites were classified as unpolluted if Igeo ≤ 0, unpolluted to moderately polluted if 0 ≤ Igeo ≤ 1, moderately polluted if 1 ≤ Igeo ≤ 2, moderate to strongly polluted if 2 ≤ Igeo ≤ 3, strongly polluted if 3 ≤ Igeo ≤ 4, strongly to extremely polluted if 4 ≤ Igeo ≤ 5, and extremely polluted if Igeo ≥ 5.

Discharges by papermills have previously been associated with pollution of the environment with PTEs, including Cr, Cd, Cu, Pb and Zn [51, 52]. This pollution can originate from a number of sources, such as metals in the wood entering the papermill, or atmospheric deposition of metals from the smoke stacks [51]. PTE-containing materials may also be used during the pulp making processes. For example, chromate bricks are used in the recovery furnace of kraft paper mills to reduce chemical attack by the spent liquors [53]. However, Cr concentrations observed in the sediments of Papermill Bay (Fig 3) are considerably lower than the 52.4 mg kg^-1^ observed in a deposit of pulp covering the sediments of a Swiss lake [54]. They are also lower than the concentrations of up to 197 mg kg^-1^ observed in a fibrous deposit in the Ångermanälven river estuary, on the east coast of Sweden [55].

The carbon and nitrogen content of the sediments at Papermill Bay was significantly (p < 0.05) and considerably greater than all of the other sites (Fig 2 and S3 Table). It was evident from visual inspection of the sediments themselves that they contained a high proportion of organic fibers (large and small strings of cellulose, and wood chips), presumably discharged into the bay from the papermill during the operational phase. The PTEs observed to be elevated in the Papermill Bay sediments (Cd, Cr, Hg and Pb) all bind strongly to organic matter in sediments [56–58]. Therefore, their presence could be due to: (i) the discharge and deposition of PTE contaminated organic material; (ii) the discharge of organic material alongside the discharge of PTEs in contaminated effluent that later bind with the organic material; or (iii) the discharge of organic material which binds to naturally present PTEs in the sediment and prevents their export to the overlying water.

In contrast to the observations made in the Papermill Bay sediments, none of the PTEs analysed in the Cassiar Cannery sediments were found to be present in significantly (p > 0.05) greater concentrations than the Inverness Passage sediments (Fig 3 and S3 Table). In fact, Co and Ni concentrations were significantly lower in Cassiar Cannery sediments than at Inverness Passage. When Inverness Passage is used as a reference site, all the PTEs analysed can be classified by the Geoaccumulation index as ‘unpolluted’ (Table 1). This observation is a clear indicator that any impact resulting from either the former use of the Cassiar Cannery site (up until 1983) as a salmon cannery, or its current use as an ecotourism lodge, can no longer be observed in the concentrations of PTEs in the top 20 cm of sediment. Any sediment contaminated by cannery waste at this site may have been buried deeper than 20 cm by fresh sediment, rendering the PTEs inaccessible to benthic invertebrates.

### Relationship between sediment properties and PTE concentrations

**Fig 4:**
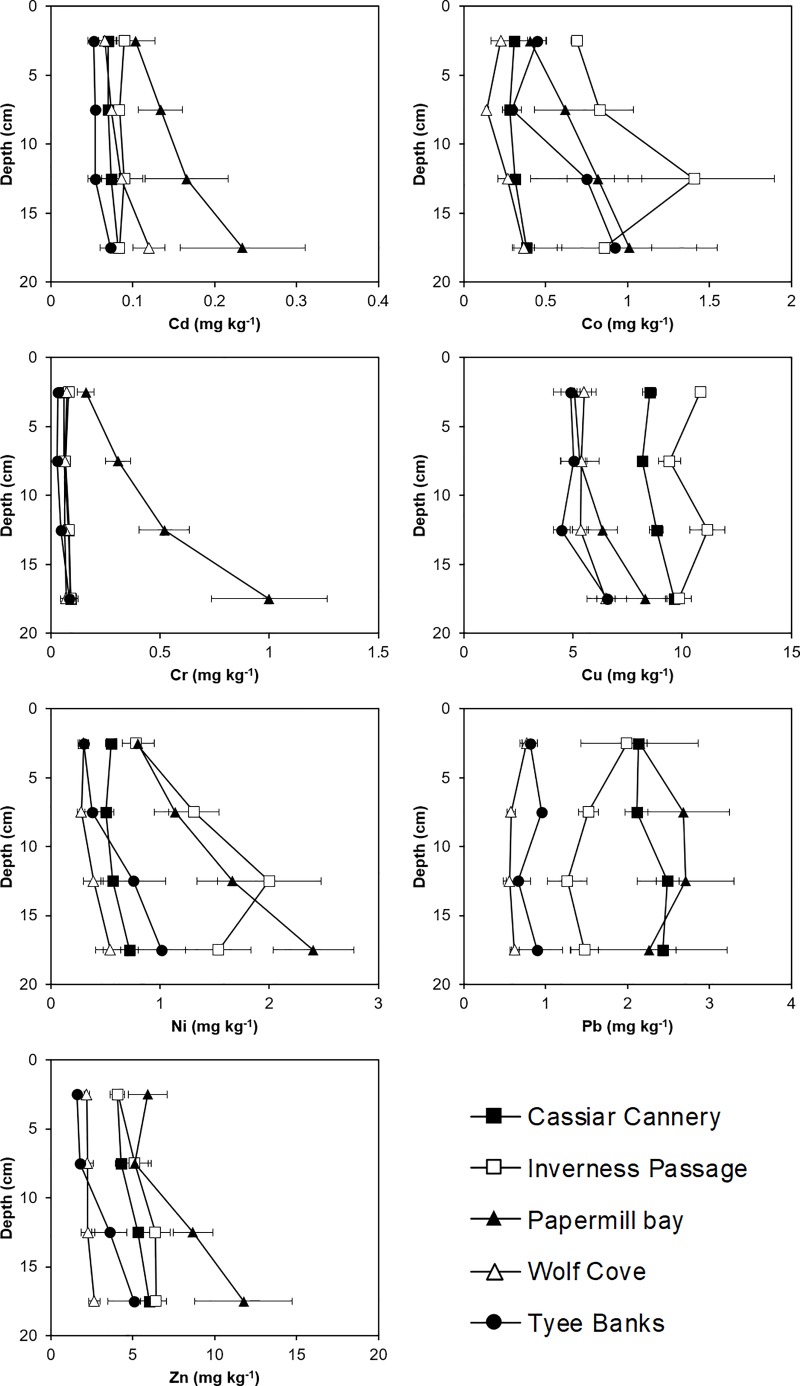
EDTA extractable (potentially bioavailable) Potentially Toxic Elements (Cd, Co, Cr, Cu, Ni, Pb and Zn) with depth in sediments sampled from Cassiar Cannery, Inverness Passage, Papermill Bay, Wolf Cove, and Tyee Banks intertidal mudflats.

Alongside greater total concentrations of Cd, Cr, Hg and Pb in the Papermill Bay sediments, compared to Wolfe Cove, we observed significantly (p < 0.05) greater EDTA extractable concentrations of Cd, Co, Cr, Ni, Pb and Zn (Fig 4 and S3 Table), indicating a greater availability of these elements to benthic invertebrates [59]. The significantly (p < 0.05) greater carbon content and the slightly finer texture of the Papermill Bay sediments, compared to Wolfe Cove (Fig 2 and S3 Table) should provide more sites for PTEs to bind to [58, 60, 61]. Thus, the greater concentrations of EDTA extractable PTEs observed was largely due to the Papermill Bay sediments being significantly (p < 0.05) more acidic than the Wolfe Cove sediments. Lower pH results in greater competition between PTEs and hydrogen ions for the binding sites on the sediment surface and leads to greater PTE lability [62, 63].

**Fig 5:**
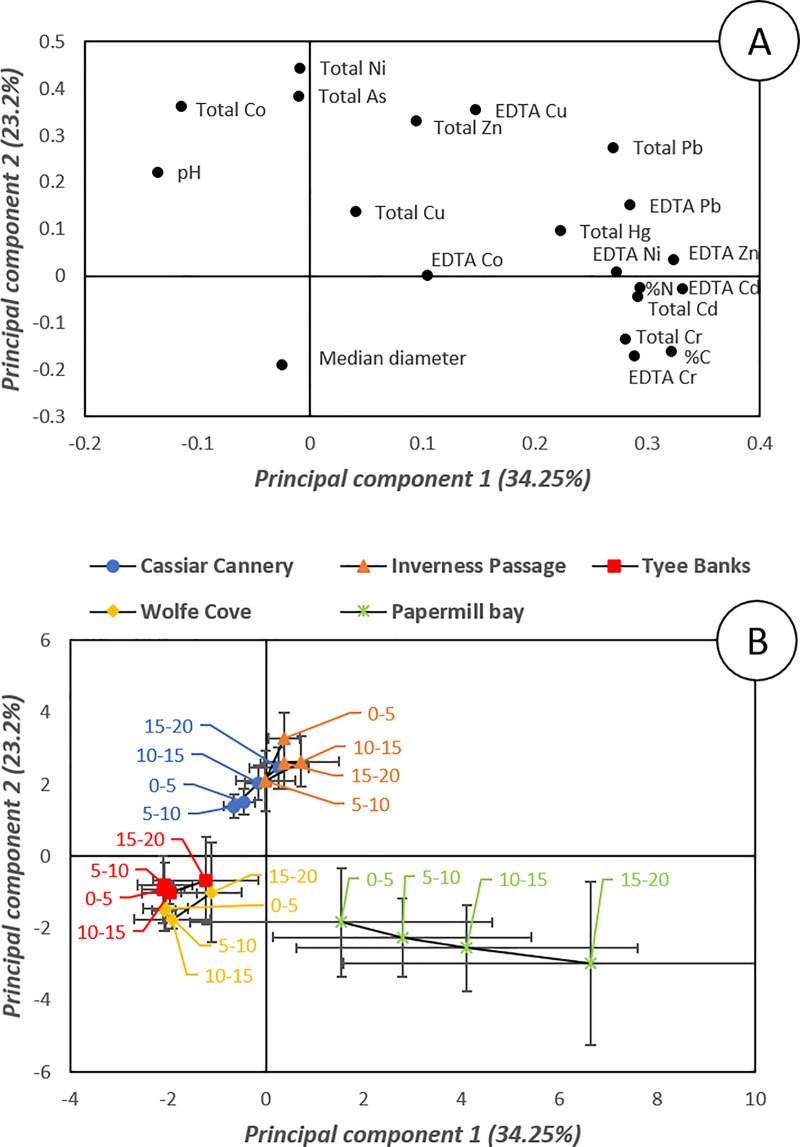
Principal components analysis of sediment properties (pH median sediment particle diameter, percentage organic carbon content and percentage total nitrogen content) and total and EDTA extractable Potentially Toxic Elements (As, Cd, Co, Cr, Cu, Hg, Ni, Pb and Zn) in sediments sampled 0–5 cm, 5–10 cm, 10–15 cm and 15-20cm from Cassiar Cannery, Inverness Passage, Papermill Bay, Wolf Cove and Tyee Banks intertidal mudflats. (A) is the latent vectors for each variable plotted in the plane of principal component one and principal component two, (B) is the principal component scores of all the samples plotted in the plane of principal component one and principal component two. Error bars represent the standard deviations of 5 replicate samples taken from 5 different transects.

The nature of the relationships between sediment properties, sediment depth and PTE concentrations were investigated. The RELATE revealed a statistically significant correlation between the concentration of PTEs and the sediment physicochemical properties resemblance matrices (Rho: 0.46; *p* = 0.001, Permutations = 9999). The relationship between total and EDTA extractable PTE concentrations and sediment properties was further explored using PCA (Fig 5). The principal component scores plot (Fig 5B) reveals a clear separation between the Papermill Bay sediments and the proximal and remote references sites of Wolfe Cove and Tyee Banks, but no separation between Cassiar Cannery and Inverness Passage, re-enforcing the conclusion that the sediments of Cassiar Cannery show little evidence of anthropogenic contamination. Principal component 2 (Fig 5), separates the Inverness Passage and Cassiar Cannery sediments from the other three sites (Papermill Bay, Wolfe Cove, and Tyee Banks), which is attributed to a different geochemical matrix composition at Cassiar Cannery and Inverness Passage, as also observed by [64]. Principal component 1 (Fig 5) reveals a separation with depth in the Papermill Bay sediments. Papermill Bay sediments have greater total and EDTA extractable concentrations of several PTEs, higher C and N, and lower pH, all of which also increased with depth in the Papermill Bay sediments. This observation of deeper layers of sediment with a lower pH and higher availability of PTEs indicates that contaminated sediment is overlain by less contaminated sediment, deposited since the discharge of sludge ceased. Although most benthic invertebrates live in surficial sediments, it is important to consider the likelihood that deeper sediment will be exposed and mobilised either by future industrial development [65], or by bioturbation from deep burrowing invertebrates [66, 67].

### Sediments host a healthy invertebrate population with no relationship between PTE concentrations in sediments and invertebrates

Overall, 22 different taxa (listed in S4 Table, along with PTE concentrations) were observed in this study, with Wolf Cove and Cassiar Cannery the most diverse sites, with 16 and 14 species collected, respectively. These taxa represent a subset of the 40 intertidal species that are commonly observed in the Skeena Estuary [5, 11]. Findings of previous studies in the Skeena Estuary reveal an infaunal invertebrate community predominantly dominated by cumaceans, polychaetes, oligochaetes, nematodes, copepods, amphipods, and bivalves [11, 68]. The 40 intertidal invertebrate species observed in this area during these previous investigations include multiple species at all trophic levels within the food web [5, 11], and such a complex community is often associated with non-disturbed habitats [21, 25]. This complexity was also observed in our study, with both predators (*Alitta brandti*, *Neries vexillosa*, *Glycinde picta*, ribbon worms and crabs) and primary consumers (*Macoma balthica*, isopods, amphipods, and sessile polychaete worms) represented [47, 69, 70]. We therefore provide evidence to support previous research indicating that the intertidal ecosystem has been passively recovering for ~50 years from past disturbances related to discharge from the papermill.

**Fig 6:**
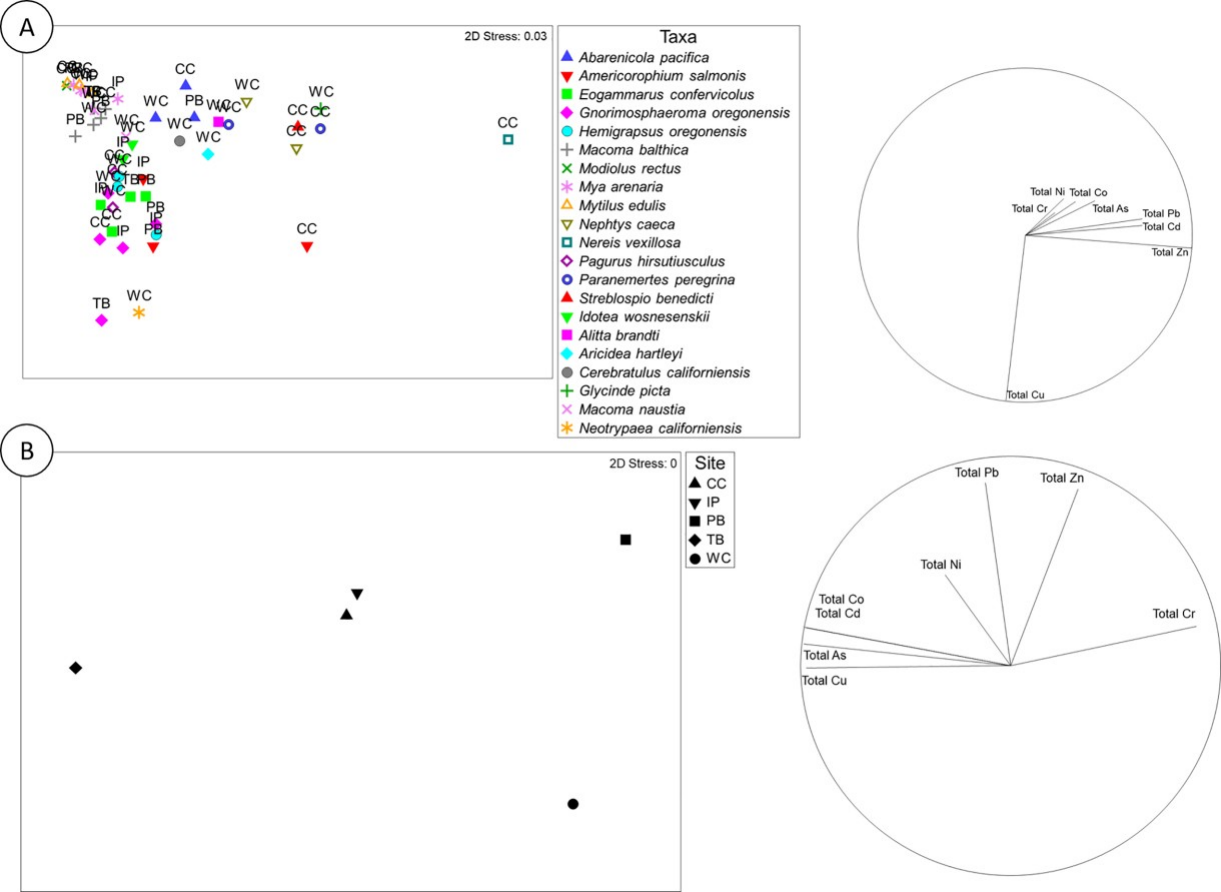
Non-metric multidimensional scaling plots (nMDS) of invertebrate PTE concentrations and the vector overlay (left hand side) of five intertidal mudflats along the north coast of British Columbia, Canada. CC: Cassiar Cannery. WC: Wolfe Cove. IP: Inverness Passage. PB: Papermill Bay. TB: Tyee Banks with (A) the entire dataset considered or (B) only including the two benthic invertebrates found at all five sites; Baltic clams (*Macoma balthica*) and Oregon pill bugs (*Gnorimosphaeroma oregonensis*).

When all invertebrates collected at all five sites are considered, we found no relationship between PTE concentrations in invertebrates and either total (Rho: 0.27; *p* = 0.32, Permutations = 9999), EDTA extractable (Rho: 0.24; *p* = 0.79, Permutations = 9999), or both total and EDTA extractable (Rho: 0.78; *p* = 0.08, Permutations = 9999) PTEs in the sediment. This finding is confirmed by inspecting a nMDS plot of invertebrate PTE loadings, which includes all organisms collected at all five sites (Fig 6A). Bivalves of the same species (e.g. *M*. *balthica*, *Mytilus edulis*, and *Mya arenaria*), collected at different sites, seem to cluster together in Fig 6A. There is slightly more separation in the PTE concentrations measured in polychaete worms (e.g. *Abarenicola pacifica*, *N*. *vexillosa*, *Paranemertes peregrina*, *G*. *picta*, *A*. *brandti*, *Nephtys caeca*, *Neotrypaea californiensis*, and *Streblospio benedicti*) which are the cause of much of the dissimilarity in the dataset.

### The suitability of *G*. *oregonensis* as a biomonitor of sediment PTE bioavailability

Selecting a benthic invertebrate to biomonitor PTE pollution requires consideration of how cosmopolitan their distribution is and the way in which they are exposed to pollutants in the environment [71]. The invertebrates sampled in this study (e.g. mussels, clams isopods, polychaete worms) acquire food using a diverse range of feeding strategies, including suspension feeding, deposit feeding at the surface or at depth, and predation of other infauna [72, 73]. Polychaete worms display all these feeding strategies. This large diversity of feeding strategies may have led to the greater range of PTE concentrations observed in Fig 6A for polychaete worms, compared to the bivalves and crustacea. Furthermore, the soft tissues of polychaete worms may only represent exposure to PTEs in the recent past due to large temporal fluctuations [74–76]. The whole body (including soft tissues and shells) of bivalves may better represent a record of time-averaged bioavailability of PTEs in the water column over the lifetime of the organism, albeit not without difficulties in interpretation [77, 78].

There were only two benthic invertebrate species observed at all five sites; Baltic clams (*M*. *balthica*) and Oregon pill bugs (*G*. *oregonensis*). When PTE concentrations in *M*. *balthica*, and *G*. *oregonensis* were compared to sediment concentrations, no relationship was observed with either total (Rho: 0.31; *p* = 0.19, Permutations = 9999), EDTA extractable (Rho: -0.0.; *p* = 0.48, Permutations = 9999), or both total and EDTA extractable (Rho: 0.21; *p* = 0.28, Permutations = 9999) sediment PTE concentrations. These findings contrast to numerous articles in the literature quantifying relationships between sediment and benthic invertebrate PTE concentrations [58, 71, 79, 80]. When we plot the PTE concentrations in *M*. *balthica*, and *G*. *oregonensis* in multidimensional space (Fig 6B), we reveal that the elemental profiles of *M*. *balthica*, and *G*. *oregonensis* contrast greatly. *M*. *Bathica* collected at Papermill Bay contained lower concentrations of Cr, Co, Ni, Zn, Cd, As and Pb than those collected at the other four sites, but higher concentrations of Cu. In contrast, *G*. *oregonensis* collected at Papermill Bay have higher concentrations of Cr, Ni, Zn, and Pb than those collected at the other four sites.

**Fig 7:**
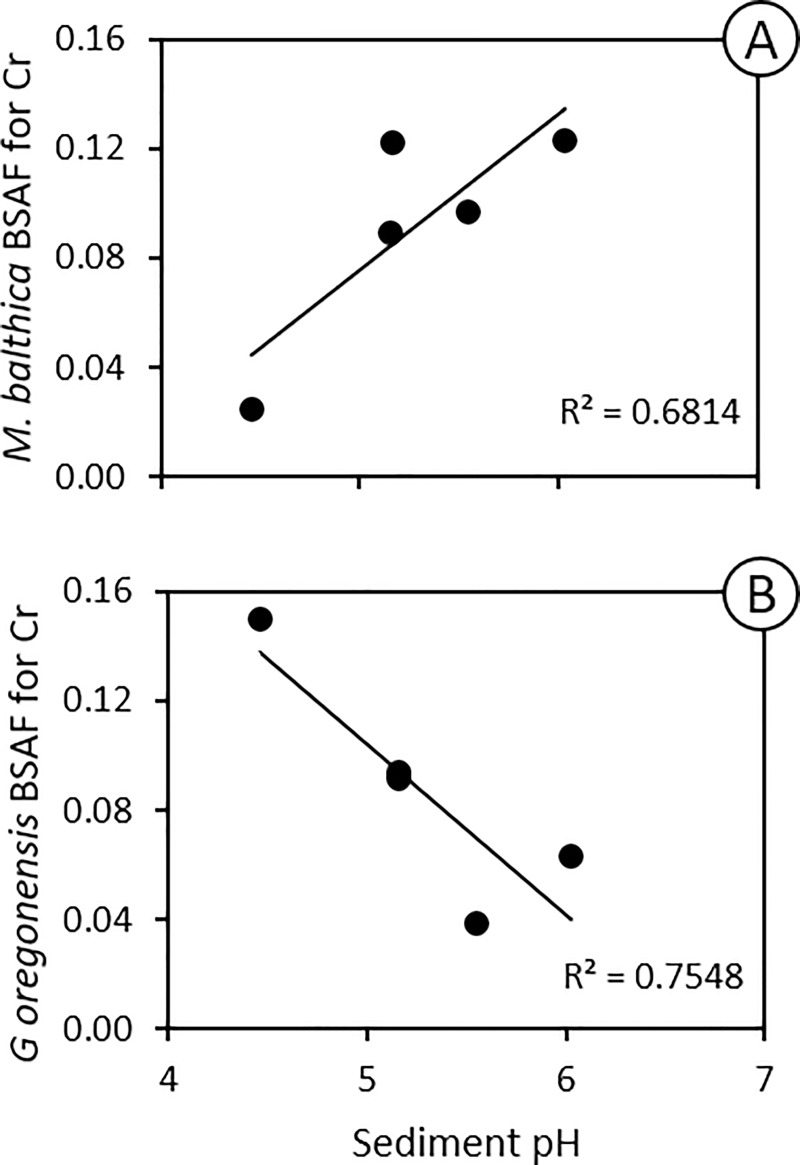
The relationship between Chromium (Cr) Biota-Sediment Accumulation Factor (BSAF) and sediment pH for (A) Baltic clams (*Macoma balthica*) and (B) Oregon pill bugs (*Gnorimosphaeroma oregonensis*) sampled at five intertidal mudflats along the north coast of British Columbia, Canada.

Relationships between sediment properties and BSAFs (calculated by dividing the concentrations of PTEs in the invertebrate tissues by the concentration in the sediments from the site from which the invertebrates were collected) reveal the importance of pH in explaining the difference in the bioaccumulation of PTEs by *M*. *balthica*, and *G*. *oregonensis*. For most PTEs there is a positive relationship between pH and the BSAF for *M Balthica* (Table 2), including a significant relationship with the Cr BSAF (Fig 7A). This is an unexpected finding that does not have an immediately obvious explanation. However, we observe a negative relationship between sediment pH and the BSAF of most PTEs for *G*. *oregonensis* (Table 2), including Cr (Fig 7B). This relationship is intuitive since metal cations dissociate from mineral surfaces at lower pH levels [62, 63]. Because *G*. *oregonensis* feeds on organic material on the surface of the sediments, the concentrations of PTEs associated with acidic sediments contaminated by papermill sludge at Papermill Bay are more likely to be assimilated by *G*. *oregonensis* and become bioaccumulated in their tissues. A survey of intertidal areas adjacent to papermills on the British Columbia coastline (including the papermill on Watson Island) identified *G*. *oregonensis* as tolerant of papermill impacted shorelines [81]. Different species of isopods have also been associated with papermill discharges on the Canadian shores of Lake Superior [82], the Swedish coast of the Gulf of Bothnia [83], and the Firth of Lome on the west coast of Scotland [84]. The greater abundance of isopods (including *G*. *oregonensis*) in close proximity to papermills, also observed by Robin, Harger [85], is attributed to the provision of pulp fibers from the mill as a food source creating a stressed environment which enables these isopods to thrive.

**Table 2:** Correlation coefficients for the relationship between sediment pH and Biota-Sediment Accumulation Factors of 8 potentially toxic elements in Baltic clams (*Macoma balthica*) and Oregon pill bugs (*Gnorimosphaeroma oregonensis*) at all five sampling locations in the Skeena Estuary.

Species	As	Cd	Co	Cr	Cu	Ni	Pb	Zn
*M Balthica*	0.4397	0.2564	0.9162	0.8255*	-0.8177	0.9461	0.5788	0.896
*G*. *oregonensis*	-0.1744	0.3758	-0.7362*	-0.8688*	-0.1895	-0.8117	-0.2083	-0.7732*

* = p < 0.05

## Conclusions

We found evidence to indicate that the discharge of papermill sludge containing PTEs from the decommissioned papermill on Watson Island has changed the sediment geochemistry at Papermill Bay by reducing the pH and increasing the total and EDTA extractable concentrations of Cd, Cr, and Pb. However, the benthic invertebrate community composition confirms that the population has mostly recovered from previous disturbance. Oregon pill bugs (*G*. *oregonensis*) were one of only two benthic invertebrate species observed at all five study sites. *G*. *oregonensis* is a cosmopolitan species and is tolerant of sites contaminated with papermill effluent because it uses the fibers discharged as a food source. Thus, we conclude here that *G*. *oregonensis* makes an excellent candidate biomonitor species to assess recovery from the environmental impact of the papermill on Watson Island and is a candidate biomonitor for monitoring the future impacts of similar industrial development projects on intertidal ecosystems.

## Supporting information

S1 File Supporting information for: Relationships between Potentially Toxic Elements in intertidal sediments and their bioaccumulation by benthic invertebrates. (PDF)

S1 Table PERMANCOVA showing sediment properties (pH, median particle diameter, C and N) varied by site, depth, and transect. (PDF)

S2 Table PERMANCOVA showing that sediment total and EDTA extractable (potentially bioavailable) PTEs varied by site and transect. (PDF)

S3 Table Analysis of Variance for sediment properties. F-statistics of a two-way ANOVA with ‘site’ and ‘depth’ as the two factors. The last two columns indicate whether reference sites; Tyee Banks (TB), Wolfe Cove (WC) and Inverness Passage (IP) are significantly (p < 0.05) different from potentially contaminated sites (Cassiar Cannery and Papermill Bay).
(PDF)

S4 Table Concentrations (mg kg^-1^) of Cr, Co, Ni, Cu, Zn, As, Cd and Pb in benthic invertebrates sampled from five intertidal mudflats along the north coast of British Columbia, Canada. CC: Cassiar Cannery. WC: Wolfe Cove. IP: Inverness Passage. PB: Papermill Bay. TB: Tyee Banks.
(PDF)

## References

[1] Gerwing TG, Plate E. Effectiveness of nutrient enhancement as a remediation or compensation strategy of salmonid fisheries in culturally oligotrophic lakes and streams in temperate climates. Restoration Ecology. 2018;27(2):278–88.

[2] Kritzer JP, DeLucia M-B, Greene E, Shumway C, Topolski MF, Thomas-Blate J, et al The Importance of Benthic Habitats for Coastal Fisheries. BioScience. 2016;66(4):274–84. 10.1093/biosci/biw014

[3] Carr-Harris C, Gottesfeld AS, Moore JW. Juvenile Salmon usage of the Skeena River Estuary. PloS One. 2015;10(3):e0118988 10.1371/journal.pone.0118988 25749488 PMC4352006

[4] Schindler DE, Scheuerell MD, Moore JW, Gende SM, Francis TB, Palen WJ. Pacific salmon and the ecology of coastal ecosystems. Frontiers in Ecology and the Environment. 2003;1(1):31–7.

[5] Gerwing TG, Gerwing AMA, Macdonald T, Cox K, Juanes F, Dudas SE. Intertidal soft-sediment community does not respond to disturbance as postulated by the intermediate disturbance hypothesis. Journal of Sea Research. 2017;129:22–8.

[6] Gerwing TG, Allen Gerwing AM, Drolet D, Barbeau MA, Hamilton DJ. Spatiotemporal variation in biotic and abiotic features of eight intertidal mudflats in the Upper Bay of Fundy, Canada. Northeastern Naturalist. 2015;22(12):1−44.

[7] Kritzer JP, DeLucia M, Greene E, Shumway C, Topolski MF, Thomas-Blate J, et al The importance of benthic habitats for coastal fisheries. BioScience. 2016;66(4):274–84.

[8] Amoozadeh E, Malek M, Rashidinejad R, Nabavi S, Karbassi M, Ghayoumi R, et al Marine organisms as heavy metal bioindicators in the Persian Gulf and the Gulf of Oman. Environmental Science and Pollution Research. 2014;21(3):2386–95. 10.1007/s11356-013-1890-8 23775003

[9] Gómez Gesteira JL, Dauvin J-C. Amphipods are good bioindicators of the impact of oil spills on soft-bottom macrobenthic communities. Marine Pollution Bulletin. 2000;40(11):1017–27.

[10] Gerwing TG, Hamilton DJ, Barbeau MA, Haralampides KA, Yamazaki G. Short-term response of a downstream marine system to the partial opening of a tidal-river causeway. Estuaries and Coasts 2017;40(3):717–25. 10.1007/s12237-016-0173-2

[11] Gerwing TG, Allen Gerwing AM, Macdonald T, Cox K, Juanes F, Dudas SE. Assessing the relationship between community dispersion and disturbance in a soft‐sediment ecosystem. Marine Ecology. 2018;39(4):e12505.

[12] Scholz B, Liebezeit G. Microphytobenthic dynamics in a Wadden Sea intertidal flat–Part II: Seasonal and spatial variability of non-diatom community components in relation to abiotic parameters. European Journal of Phycology. 2012;47(2):120–37.

[13] van Proosdij D, Milligan T, Bugden G, Butler K. A tale of two macro tidal estuaries: differential morphodynamic response of the intertidal zone to causeway construction. Journal of Coastal Research. 2009;56:772−6.

[14] Pourret O, Bollinger J-C. “Heavy metal”—What to do now: To use or not to use? Science of The Total Environment. 2018;610–611:419–20. 10.1016/j.scitotenv.2017.08.043 28810151

[15] Majer AP, Petti MAV, Corbisier TN, Ribeiro AP, Theophilo CYS, de Lima Ferreira PA, et al Bioaccumulation of potentially toxic trace elements in benthic organisms of Admiralty Bay (King George Island, Antarctica). Marine pollution bulletin. 2014;79(1–2):321–5. 10.1016/j.marpolbul.2013.12.015 24368117

[16] Wang X, Yang H, Gong P, Zhao X, Wu G, Turner S, et al One century sedimentary records of polycyclic aromatic hydrocarbons, mercury and trace elements in the Qinghai Lake, Tibetan Plateau. Environmental Pollution. 2010;158(10):3065–70. 10.1016/j.envpol.2010.06.034 20650556

[17] de Souza Machado AA, Spencer K, Kloas W, Toffolon M, Zarfl C. Metal fate and effects in estuaries: A review and conceptual model for better understanding of toxicity. Science of The Total Environment. 2016;541:268–81. 10.1016/j.scitotenv.2015.09.045 26410702

[18] Moore JW, Gordon J, Carr-Harris C, Gottesfeld AS, Wilson SM, Russell JH. Assessing estuaries as stopover habitats for juvenile Pacific salmon. Marine Ecology Progress Series. 2016;559:201–15.

[19] Tuominen TM, Sekela MA. Dioxins and furans in sediment and fish from the vicinity of four inland pulp and/or paper mills and one petroleum refinery in British Columbia: Environment Canada. Conservation and Protection; 1992.

[20] Wilkes B, Dwernychuk LW. Environment studies in the marine receiving environment at the Skeena Cellulose pulp mill, Watson Island, BC. Pulp & Paper Canada. 1991:92:10.

[21] Pearson TH, Rosenberg R. A comparative study of the effects on the marine environment of wastes from cellulose industries in Scotland and Sweden. Ambio. 1976:77–9.

[22] Waldichuk M, Bousfield EL. Amphipods in low-oxygen marine waters adjacent to a sulphite pulp mill. Journal of the Fisheries Board of Canada. 1962;19(6):1163–5.

[23] Gerwing TG. Preliminary report of intertidal research along the north coast of British Columbia: Summer 2016. Report to the Kitsumkalum First Nations. 34 p. 2016.

[24] Gerwing TG, Cox K, Gerwing AMA, Carr-Harris CN, Dudas SE, Juanes F. Depth to the apparent redox potential discontinuity (aRPD) as a parameter of interest in marine benthic habitat quality models. International Journal of Sediment Research. 2018;33(2):149–56.

[25] Pearson TH, Rosenberg R. Macrobenthic succession in relation to organic enrichment and pollution of the marine environment. Oceanography and Marine Biology: An Annual Review. 1978;16:229–331.

[26] Allen L. The application of biodiversity indicators to infer ecosystem health in regenerating tropical forest: University of Glasgow; 2019.

[27] Zhang W, Dulloo E, Kennedy G, Bailey A, Sandhu H, Nkonya E. Biodiversity and Ecosystem Services Sustainable Food and Agriculture: Elsevier; 2019 p. 137–52.

[28] Smith MD, Knapp AK. Exotic plant species in a C 4-dominated grassland: invasibility, disturbance, and community structure. Oecologia. 1999;120(4):605–12. 10.1007/s004420050896 28308312

[29] Burke MJW, Grime JP. An experimental study of plant community invasibility. Ecology. 1996;77(3):776–90.

[30] Guerra-García JM, García-Gómez JC. Polychaete assemblages and sediment pollution in a harbour with two opposing entrances. Helgoland Marine Research. 2004;58(3):183–91.

[31] Gerwing TG, Campbell L, Allen Gerwing AM, Allen S, Cox K, Rogers M, et al Potential impacts of logging on intertidal infaunal communities within the Kitimat River estuary. Journal of Natural History. 2018;52(43–44):2833–55.

[32] HC. Hatfield Consultants Ltd. Skeena environmental effects monitoring (EEM) pre-design reference document. Prepared for Skeena Cellulose Inc. 1994.

[33] Hoos LM. The Skeena River Estuary: status of environmental knowledge to 1975: Report of the Estuary Working Group, Department of the Environment, Regional Board, Pacific Region. Environment Canada, 1975 Contract No.: 3.

[34] NIBR. Northwest Institute for Bioregional Research: Valuation of the wild Salmon economy of the Skeena River watershed. 1–30 2006.

[35] Hilborn R, Walters CJ. Differing goals of salmon management on the Skeena River. Journal of the Fisheries Board of Canada. 1977;34(1):64–72.

[36] Higgins RJ, Schouwenburg WJ. A biological assessment of fish utilization of the Skeena River Estuary, with special reference to port development in Prince Rupert Technical Report 1973–1. Department of Environment, Fisheries and Marine Services Vancouver, BC, 1973.

[37] Faggetter BA. Review of the Environmental and Socioeconomic Impacts of Marine Pollution in the North and Central Coast Regions of British Columbia. 2008.

[38] Yunker MB, Cretney WJ, Ikonomou MG. Assessment of chlorinated dibenzo-p-dioxin and dibenzofuran trends in sediment and crab hepatopancreas from pulp mill and harbor sites using multivariate- and index-based approaches. Environmental Science & Technology. 2002;36(9):1869–78. 10.1021/es0112893 WOS:000175311900006. 12026964

[39] Akenhead S. A review of the oceanography and marine ecology of Prince Rupert Harbour a propos sewage outfalls. 1992.

[40] Cox K, Black M, Filip N, Miller M, Mohns K, Mortimor J, et al Comparison of community assessment techniques and implications for diversity indices and species accumulation curves. Ecology and Evolution. 2017; 10.1002/ece3.3580 PMC5743490 29299294

[41] Gerwing TG, Gerwing AMA, Cox K, Juanes F, Dudas SE. Relationship between apparent redox potential discontinuity (aRPD) depth and environmental variables in soft-sediment habitats. International Journal of Sediment Research. 2017;32(4):472–80.

[42] BS7755-3.2. Soil Quality Part 3: Chemical Methods. Section 3.2: Determination of pH British Standards Institution, London, UK 1995.

[43] EPA US. Method 3051A (SW-846): Microwave Assisted Acid Digestion of Sediments, Sludges, and Oils, Revision 1. Washington, DC. 2007.

[44] EPA US. Method 7473 (SW-846): Mercury in Solids and Solutions by Thermal Decomposition, Amalgamation, and Atomic Absorption Spectrophotometry, Revision 0. Washington, DC. 1998.

[45] Muller G. Index of geoaccumulation in sediments of the Rhine River. Geojournal. 1969;2:108–18.

[46] Anderson M, Gorley RN, Clarke RK. Permanova+ for Primer: Guide to software and statistical methods. Plymouth, United Kingdom: PRIMER-E Ltd; 2008.

[47] Gerwing TG, Drolet D, Hamilton DJ, Barbeau MA. Relative importance of biotic and abiotic forces on the composition and dynamics of a soft-sediment intertidal community PLoS One. 2016;11(1):11:e0147098. 10.1371/journal.pone.0147098 PMC4720360 26790098

[48] Clarke KR, Gorley RN. PRIMER v7: user manual/tutorial 3rd ed. Plymouth, United Kingdom: Primer-E Ltd; 2015.

[49] McLaren P. The environmental implications of sediment transport in the waters of Prince Rupert, British Columbia, Canada: A comparison between kinematic and dynamic approaches. Journal of Coastal Research. 2016;32(3):465–82.

[50] Monte MC, Fuente E, Blanco A, Negro C. Waste management from pulp and paper production in the European Union. Waste management. 2009;29(1):293–308. 10.1016/j.wasman.2008.02.002 18406123

[51] Hoffman E, Lyons J, Boxall J, Robertson C, Lake CB, Walker TR. Spatiotemporal assessment (quarter century) of pulp mill metal (loid) contaminated sediment to inform remediation decisions. Environmental monitoring and assessment. 2017;189(6):257 10.1007/s10661-017-5952-0 28478542

[52] Borah P, Singh P, Rangan L, Karak T, Mitra S. Mobility, bioavailability and ecological risk assessment of cadmium and chromium in soils contaminated by paper mill wastes. Groundwater for Sustainable Development. 2018;6:189–99.

[53] Nriagu JO, Nieboer E. Chromium in the natural and human environments: John Wiley & Sons; 1988.

[54] Kienle C, Langer-Jaesrich M, Baumberger D, Hohmann D, Santiago S, Köhler H-R, et al Integrated toxicity evaluation of a pulp deposit using organisms of different trophic levels. Journal of soils and sediments. 2013;13(9):1611–25.

[55] Apler A, Snowball I, Frogner-Kockum P, Josefsson S. Distribution and dispersal of metals in contaminated fibrous sediments of industrial origin. Chemosphere. 2019;215:470–81. 10.1016/j.chemosphere.2018.10.010 30340155

[56] Alvarez MB, Malla ME, Batistoni DA. Comparative assessment of two sequential chemical extraction schemes for the fractionation of cadmium, chromium, lead and zinc in surface coastal sediments. Fresenius' journal of analytical chemistry. 2001;369(1):81–90. 11210236 10.1007/s002160000592

[57] Chakraborty P, Babu PVR, Sarma VV. A study of lead and cadmium speciation in some estuarine and coastal sediments. Chemical Geology. 2012;294:217–25.

[58] Sizmur T, Canário J, Gerwing TG, Mallory ML, O'Driscoll NJ. Mercury and methylmercury bioaccumulation by polychaete worms is governed by both feeding ecology and mercury bioavailability in coastal mudflats. Environmental Pollution. 2013;176:18–25. 10.1016/j.envpol.2013.01.008 23395989

[59] Weimin Y, Batley G, Ahsanullah M. The ability of sediment extractants to measure the bioavailability of metals to three marine invertebrates. Science of the Total Environment. 1992;125:67–84.

[60] Burton ED, Phillips IR, Hawker DW. Geochemical partitioning of copper, lead, and zinc in benthic, estuarine sediment profiles. Journal of environmental quality. 2005;34(1):263–73. 15647557 10.2134/jeq2005.0263

[61] Luoma SN. Processes affecting metal concentrations in estuarine and coastal marine sediments Heavy metals in the marine environment: CRC Press; 2017 p. 51–66.

[62] Riba I, Delvalls TÁ, Forja JM, Gómez‐Parra A. The influence of pH and salinity on the toxicity of heavy metals in sediment to the estuarine clam Ruditapes philippinarum. Environmental toxicology and chemistry. 2004;23(5):1100–7. 15180359 10.1897/023-601

[63] Zhang C, Yu Z-g, Zeng G-m, Jiang M, Yang Z-z, Cui F, et al Effects of sediment geochemical properties on heavy metal bioavailability. Environment International. 2014;73:270–81. 10.1016/j.envint.2014.08.010 25173943

[64] DelValls TÁ, Forja J, González-Mazo E, Gómez-Parra A, Blasco J. Determining contamination sources in marine sediments using multivariate analysis. TrAC Trends in Analytical Chemistry. 1998;17(4):181–92.

[65] Chapman PM, Anderson J. A decision‐making framework for sediment contamination. Integrated environmental assessment and management. 2005;1(3):163–73. 16639882 10.1897/2005-013r.1

[66] Ciutat A, Boudou A. Bioturbation effects on cadmium and zinc transfers from a contaminated sediment and on metal bioavailability to benthic bivalves. Environmental toxicology and chemistry. 2003;22(7):1574–81. 12836984

[67] Sizmur T, Canário J, Edmonds S, Godfrey A, O'Driscoll NJ. The polychaete worm Nereis diversicolor increases mercury lability and methylation in intertidal mudflats. Environmental Toxicology and Chemistry. 2013;32(8):1888–95. 10.1002/etc.2264 23633443

[68] Gerwing TG, Allen Gerwing AM, Macdonald T, Cox K, Juanes F, Dudas SE. Intertidal soft-sediment community does not respond to disturbance as postulated by the intermediate disturbance hypothesis. Journal of Sea Research. 2017;129:22–8.

[69] Light SF. The Light and Smith manual: intertidal invertebrates from central California to Oregon. 4 ed. Cameron JT, editor: University of California Press, Berkely, CA, USA; 2007.

[70] Gerwing TG, Kim JH, Hamilton DJ, Barbeau MA, Addison JA. Diet reconstruction using next-generation sequencing increases the known ecosystem usage by a shorebird. The Auk. 2016;133(2):168−77.

[71] Rainbow PS. Biomonitoring of heavy metal availability in the marine environment. Marine pollution bulletin. 1995;31(4–12):183–92.

[72] Fauchald K, Jumars PA. The diet of worms: a study of polychaete feeding guilds. Oceanography and Marine Biology Annual Review. 1979;17:193–284.

[73] Pagliosa PR. Another diet of worms: the applicability of polychaete feeding guilds as a useful conceptual framework and biological variable. Marine Ecology. 2005;26:246–54.

[74] Howard L, Brown B. Natural variations in tissue concentration of copper, zinc and iron in the polychaete Nereis diversicolor. Marine biology. 1983;78(1):87–97.

[75] Saha M, Sarkar S, Bhattacharya B. Interspecific variation in heavy metal body concentrations in biota of Sunderban mangrove wetland, northeast India. Environment International. 2006;32(2):203–7. 10.1016/j.envint.2005.08.012 16213017

[76] Saiz‐Salinas J, Francés‐Zubillaga G. Nereis diversicolor: an unreliable biomonitor of metal contamination in the ‘Ría de Bilbao’(Spain). Marine Ecology. 1997;18(2):113–25.

[77] Schöne BR, Krause RA Jr. Retrospective environmental biomonitoring–Mussel Watch expanded. Global and Planetary Change. 2016;144:228–51.

[78] Zuykov M, Pelletier E, Harper DAT. Bivalve mollusks in metal pollution studies: from bioaccumulation to biomonitoring. Chemosphere. 2013;93(2):201–8. 10.1016/j.chemosphere.2013.05.001 23751124

[79] Caçador I, Costa J, Duarte B, Silva G, Medeiros J, Azeda C, et al Macroinvertebrates and fishes as biomonitors of heavy metal concentration in the Seixal Bay (Tagus Estuary): which species perform better? Ecological Indicators. 2012;19:184–90.

[80] Sarkar SK, Cabral H, Chatterjee M, Cardoso I, Bhattacharya AK, Satpathy KK, et al Biomonitoring of heavy metals using the bivalve molluscs in Sunderban mangrove wetland, northeast coast of Bay of Bengal (India): possible risks to human health. CLEAN–Soil, Air, Water. 2008;36(2):187–94.

[81] Bard S M,. A biological index to predict pulp mill pollution levels. Water environment research. 1998;70(1):108–22.

[82] Sibley PK, Dixon DG, Barton DR. Impact of bleached kraft pulp mill effluent on benthic community structure in relation to environmental factors. Journal of Aquatic Ecosystem Stress and Recovery. 2000;7(3):229–46. 10.1023/A:1009987123319

[83] Leonardsson K. Long-term ecological effects of bleached pulp-mill effluents on benthic macrofauna in the Gulf of Bothnia. Ambio. 1993:359–62.

[84] Pearson T. The effect of industrial effluent from pulp and paper mills on the marine benthic environment. Proceedings of the Royal Society of London Series B Biological Sciences. 1972;180(1061):469–85. 10.1098/rspb.1972.0032 4402205

[85] Robin J, Harger E, Nassichuk MD. Marine intertidal community responses to Kraft pulp mill effluent. Water, Air, and Soil Pollution. 1974;3(1):107–22.

