# Supplementary material for: Relationships between Potentially Toxic Elements in intertidal sediments and their bioaccumulation by benthic invertebrates
Source: PLoS One. 2019 Sep 19;14(9):e0216767. doi: 10.1371/journal.pone.0216767 (PMC6752810; doi:10.1371/journal.pone.0216767)
Supplement: S1 File — (PDF) [file pone.0216767.s001.pdf]

Supporting Information for:

Relationships between Potentially Toxic Elements in intertidal sediments and their bioaccumulation by benthic invertebrates

Tom Sizmur[1], Lily Campbell[2], Karina Dracott[3], Megan Jones[1], Nelson J. O'Driscoll[4] and Travis Gerwing[2,5,6]

Quality control

Total organic carbon and nitrogen

Analysis of sediment samples for total organic carbon and nitrogen content using a Thermo Scientific Flash 2000 Organic Elemental Analyser was conducted alongside one blank every 20 samples and a 5 mg in-house reference soil (107% ± 10.08% recovery for N and 101% ± 0.99% recovery for C, n = 4) traceable to GBW07412, certified for N by State Bureau of Technical Supervision, The People's Republic of China and to AR-4016, certified for C by Alpha Resources Inc.. The instrument was calibrated with 1 and 3 mg samples of an aspartic acid standard.

ICP-MS analysis of sediment digests

Each batch of 33 samples was digested alongside four blank tubes, two samples of an in-house reference soil (As: 110% ± 6.58%, Cd: 95% ± 4.01%, Co: 81% ± 18.24%, Cr: 91% ± 5.33%, Cu: 88% ± 6.16%, Ni: 79% ± 2.90%, Pb: 97% ± 8.98%, Zn: 104% ± 10.91%, n = 6) traceable to BCR-143R trace elements in a sewage sludge amended soil, certified by Commission of the European Communities, Community Bureau of Reference, and one sample of BCR - 320R Channel Sediment, certified by Commission of the European Communities, Community Bureau of Reference (As: 104% ± 2.44%, Cd: 100% ± 3.16%, Co: 87% ± 3.55%, Cr: 46% ± 2.39%, Cu: 94% ± 4.49%, Ni: 81% ± 3.45%, Pb: 102% ± 4.04%, Zn: 98% ± 2.85%, n = 3). The BCR-320R Channel Sediment is certified for total concentrations, whereas the method adopted here (U.S. EPA Method 3051A) is not intended to accomplish total decomposition of the sample.

Mercury analysis of sediments

The sediments were measured alongside three samples of BCR-320R Channel Sediment, certified by the Commission of the European Communities, Community Bureau of Reference (92% ± 3.03, n = 3) and nine samples of DORM-4 Dogfish Muscle, certified by the National Research Council, Canada (92% ± 2.32, n = 9).

ICP-MS analysis of invertebrate digests

The samples were analysed in two batches, each alongside six blank tubes and two samples of ERM-CE278 Mussel tissue; Commission of the European Communities, Community Bureau of Reference (As:

104% ± 11.10%, Cd: 90% ± 4.78%, Cr: 114% ± 5.74%, Cu: 77% ± 13.39%, Pb: 106% ± 5.54%, Zn: 83% ± 10.19%, n = 4).

Statistical analysis

Effect of site and sediment depth on sediment properties and PTE concentrations

The influence of site and sediment depth on sediment PTE concentrations and properties was quantified using two-way analysis of variance using the 18th Edition of Genstat. Normality and homoscedasticity were assessed by inspecting the residual plots and logarithmic or reciprocal transformations made, where necessary. Multiple comparisons were made using the Fisher Least Significant Differences test at the 95% level. Spatial variation of sediment properties (C, N, pH, and median diameter) was assessed using permutational multivariate analysis of covariance, PERMANCOVA (Anderson et al. 2008; Gerwing et al. 2016a). The response variable for the PERMANCOVA was a resemblance matrix calculated using Euclidian distances, and sediment properties were normalized prior to analysis. Before normalization, all variables were square root transformed to correct for skewed distributions (Clarke 1993; Clarke et al. 2008). In the PERMANOVA, site (five levels) was a fixed factor and transect nested within site (5 levels) was a random factor. Depth was included as a covariate. The lowest level of replication was an individual sediment core, n = 76 seines. As part of the PERMANCOVA, we quantified variance components, the proportion of the multivariate variation accounted for by each variable (Searle et al. 1992; Anderson et al. 2008).

The relationship between PTE concentrations and sediment properties

The relationship between PTE concentrations and sediment properties was tested in a step-wise manner. First, using the program PRIMER (Clarke & Gorley 2015), the relationship at all sediment depths, was quantified using PRIMER's RELATE function. This function compares two resemblance matrices looking for any relationships. In this case, the resemblance matrices were composed of sediment physicochemical property data and sediment PTE data. The sediment physicochemical property resemblance matrix was composed of four variables (pH, %N, %C, and median particle size), all of which were fourth root transformed to correct for skewed distributions. Sediment physicochemical property data were then normalized and a resemblance matrix was constructed from these data using Euclidian distances. The sediment PTE resemblance matrix was constructed in the same manner, except data did not require a transformation to correct for skewed distributions, but data were normalized. As a significant relationship was observed between the two resemblances matrices, this relationship was further explored using Principal Component Analyses on the variance-covariance matrix of all sediment PTE and sediment property data using the 18th edition of Genstat. A PERMANCOVA, conducted as described above, was also used to test the spatial variation in total and extractable PTE concentrations in sediment (n = 98).

The relationship between PTE concentrations in sediments and invertebrates

Relationships between the PTE concentrations observed in the sediments and in the collected invertebrates were examined in several configurations using RELATE, and in all cases the sediment PTE resemblance matrix was constructed as described above on normalized data. First, a sediment PTE resemblance matrix was compared to the observed concentrations of all metals identified in invertebrates, (total and extractable), as well as total and extractable metals analyzed separately.

Finally, as not all invertebrates were observed at all sites, we looked for a relationship between contamination (all PTEs, total, and extractable) in two invertebrates observed at each site (*M. balthica*, and *G. oregonensis*) and PTEs in the sediment. For all the analyses described above, all depth levels were retained in the resemblance matrices. When relationships between sediment and invertebrate contaminations were separated by depths, similar relationships were observed. As such, we only present relationships that include all depths here. Non-metric multidimensional (nMDS) scaling plots were used to assess relationships between invertebrates and PTE concentrations. The response variable for all nMDS plots were the resemblance matrices described above, and nMDS plot construction incorporated 100 restarts. All nMDS graphs had a stress ~0.2, and were considered good 2-dimensonal representation of higher dimensional trends (Clarke 1993).
