# Supplementary material for: Relationships between Potentially Toxic Elements in intertidal sediments and their bioaccumulation by benthic invertebrates
Source: PLoS One. 2019 Sep 19;14(9):e0216767. doi: 10.1371/journal.pone.0216767 (PMC6752810; doi:10.1371/journal.pone.0216767)
Supplement: S1 Table — (PDF) [file pone.0216767.s002.pdf]

S1 Table. PERMANCOVA showing sediment properties (pH, median particle diameter, C and N) varied by site, depth, and transect.

Source	df	MS	Pseudo-F	Unique Permutations	*p*	Variance Components (%)
Depth	1	2.23	6.44	9943	0.0006	1.52
Site	4	13.17	9.15	9935	0.0001	50.24
Transect (site)	16	1.66	5.51	9887	0.0001	22.41
Depth X Site	3	1.28	4.25	9954	0.0004	3.35
Depth X Transect (Site)	16	0.53	1.76	9899	0.02	3.97
Residual	35	0.30				18.52
Total	75					
