# Supplementary material for: Relationships between Potentially Toxic Elements in intertidal sediments and their bioaccumulation by benthic invertebrates
Source: PLoS One. 2019 Sep 19;14(9):e0216767. doi: 10.1371/journal.pone.0216767 (PMC6752810; doi:10.1371/journal.pone.0216767)
Supplement: S2 Table — (PDF) [file pone.0216767.s003.pdf]

S2 Table. PERMANCOVA showing that sediment total and available (EDTA extractable) PTEs varied by site and transect.

Source	df	MS	Pseudo-F	Unique Permutations	*p*	Variance Components (%)
Depth	1	572.5	1.48	9945	0.23	0.18
Site	4	11480.0	14.62	9945	0.0001	50.88
Transect(Site)	20	781.0	2.04	9902	0.002	9.50
Depth X Site	4	409.8	1.07	9937	0.39	0.13
Depth X Transect(Site)	20	535.1	1.40	9906	0.13	3.71
Residual	48	382.0				35.60
Total	97					
