# Supplementary material for: Relationships between Potentially Toxic Elements in intertidal sediments and their bioaccumulation by benthic invertebrates
Source: PLoS One. 2019 Sep 19;14(9):e0216767. doi: 10.1371/journal.pone.0216767 (PMC6752810; doi:10.1371/journal.pone.0216767)
Supplement: S3 Table — The last two columns indicate whether reference sites; Tyee Banks (TB), Wolfe Cove (WC) and Inverness Passage (IP) are significantly (p < 0.05) different from potentially contaminated sites (Cassiar Cannery and Papermill Bay). (PDF) [file pone.0216767.s004.pdf]

S3 Table. Analysis of Variance for sediment properties. F-statistics of a two-way ANOVA with 'site' and 'depth' as the two factors. The last two columns indicate whether reference sites; Tyee Banks (TB), Wolfe Cove (WC) and Inverness Passage (IP) are significantly ($p < 0.05$) different from potentially contaminated sites (Cassiar Cannery and Papermill Bay).

Variable		Site	Depth	Site × Depth interaction	Cassiar Cannery	Papermill Bay
Sediment pH		24.96***	1.39	1.27	IP, TB	WC, TB
Median sediment diameter	#	53.92***	0.85	1.97*	TB	TB
Sediment %C	$	49.82***	3.67*	1.35	TB	WC, TB
Sediment %N	$	41.00***	1.67	0.53	TB	WC, TB
Total sediment As		183.99***	2.54	1.1	IP	WC, TB
Total sediment Cd	$	11.49***	1.28	0.8	TB	TB
Total sediment Co		76.88***	2.09	1.93*	IP, TB	WC, TB
Total sediment Cr	$	75.73***	0.17	0.73	TB	WC, TB
Total sediment Cu		61.51***	3.45*	0.66	TB	WC, TB
Total sediment Hg	#	41.91***	0.13	0.77	TB	WC, TB
Total sediment Ni		108.17***	1.36	0.5	IP, TB	WC, TB
Total sediment Pb	$	89.15***	0.43	0.53	TB	WC, TB
Total sediment Zn		14.27***	1.47	0.79	TB	TB
EDTA extractable sediment Cd	$	8.19***	2.62	0.47	TB	WC, TB
EDTA extractable sediment Co	$	17.72***	4.45**	1.63	IP	WC
EDTA extractable sediment Cr	#	72.22***	11.22***	2.67**	TB	WC, TB
EDTA extractable sediment Cu		45.67***	5.43**	1.31	IP, TB	TB
EDTA extractable sediment Ni		21.64***	10.24***	1.86	IP	WC, TB
EDTA extractable sediment Pb		22.97***	0.01	0.5	IP, TB	WC, TB
EDTA extractable sediment Zn		20.63***	9.67***	1.31	TB	WC, TB

# = Log_{10} transformed, $ = Reciprocal transformed, * = P< 0.05, ** = P< 0.01, *** = P< 0.001
