# Supplementary material for: Relationships between Potentially Toxic Elements in intertidal sediments and their bioaccumulation by benthic invertebrates
Source: PLoS One. 2019 Sep 19;14(9):e0216767. doi: 10.1371/journal.pone.0216767 (PMC6752810; doi:10.1371/journal.pone.0216767)
Supplement: S4 Table — CC: Cassiar Cannery. WC: Wolfe Cove. IP: Inverness Passage. PB: Papermill Bay. TB: Tyee Banks. (PDF) [file pone.0216767.s005.pdf]

S4 Table. Concentrations (mg kg^{-1}) of Cr, Co, Ni, Cu, Zn, As, Cd and Pb in benthic invertebrates sampled from five intertidal mudflats along the north coast of British Columbia, Canada. CC: Cassiar Cannery. WC: Wolfe Cove. IP: Inverness Passage. PB: Papermill Bay. TB: Tyee Banks

Site	Common name	Latin name	As	Cd	Co	Cr	Cu	Ni	Pb	Zn
CC	Clams	*Macoma balthica*	6.25	0.05	1.67	3.72	20.59	3.38	0.90	39.92
IP	Clams	*Macoma balthica*	4.59	0.07	1.76	3.69	21.11	2.39	0.95	30.42
TB	Clams	*Macoma balthica*	7.59	0.07	1.24	2.17	20.80	1.50	0.55	29.24
WC	Clams	*Macoma balthica*	3.19	0.04	1.49	3.76	34.60	1.99	0.70	30.73
PB	Clams	*Macoma balthica*	2.09	0.03	0.71	1.14	44.49	0.98	0.27	16.62
CC	Blue mussels	*Mytilus edulis*	2.16	0.44	0.89	1.45	3.44	0.98	0.23	8.01
WC	Blue mussels	*Mytilus edulis*	5.00	0.82	0.66	0.99	3.71	1.01	0.53	18.13
IP	Blue mussels	*Mytilus edulis*	6.43	1.26	3.79	4.60	9.50	3.10	1.04	27.75
CC	Horse mussels	*Modiolus rectus*	1.74	0.29	0.88	2.30	5.67	1.36	0.67	6.91
WC	Soft shell clams	*Mya arenaria*	4.22	0.20	2.72	7.43	9.36	3.21	1.11	21.14
PB	Soft shell clams	*Mya arenaria*	2.43	0.12	1.05	1.48	3.70	0.87	0.72	13.96
CC	Soft shell clams	*Mya arenaria*	6.80	0.22	2.21	4.29	8.26	3.49	1.01	18.38
IP	Soft shell clams	*Mya arenaria*	15.61	0.39	6.41	6.41	13.36	4.93	1.83	47.16
WC	Bent nose macoma	*Macoma naustia*	3.82	0.11	2.78	6.82	22.97	3.64	1.00	30.52
WC	Bent nose macoma	*Macoma naustia*	5.98	0.19	4.45	10.38	42.02	4.96	1.49	57.06
CC	Green shore crabs	*Hemigrapsus oregonensis*	7.28	0.25	1.76	3.15	82.84	2.45	0.76	53.81
IP	Green shore crabs	*Hemigrapsus oregonensis*	8.91	0.34	1.68	2.59	116.89	2.19	0.61	88.73
WC	Green shore crabs	*Hemigrapsus oregonensis*	6.05	0.27	0.71	1.33	74.81	1.17	0.76	54.80
WC	Hermit crabs	*Pagurus hirsutiusculus*	5.55	0.21	1.46	3.99	100.06	2.59	1.31	51.29
CC	Hermit crabs	*Pagurus hirsutiusculus*	4.01	0.26	0.34	0.48	71.57	0.38	0.14	50.78
CC	Isopods	*Gnorimosphaeroma oregonensis*	4.52	0.41	1.68	1.91	125.23	1.59	0.43	43.51
TB	Isopods	*Gnorimosphaeroma oregonensis*	5.55	0.67	2.61	2.82	186.78	2.30	0.64	50.52
WC	Isopods	*Gnorimosphaeroma oregonensis*	3.37	0.20	1.11	2.23	88.58	1.23	0.34	47.07
IP	Isopods	*Gnorimosphaeroma oregonensis*	5.47	0.46	1.58	1.48	130.14	1.33	0.48	63.75
PB	Isopods	*Gnorimosphaeroma oregonensis*	5.42	0.36	2.54	6.96	109.07	3.38	0.81	87.51

WC	Kelp isopods	*Idotea wosnesenskii*	6.54	0.43	1.62	2.54	47.23	2.10	0.68	63.27
IP	Kelp isopods	*Idotea wosnesenskii*	8.85	0.52	1.96	1.28	60.00	1.88	0.42	56.48
WC	Ghost shrimp	*Neotrypaea californiensis*	14.30	1.32	1.79	1.89	177.56	1.16	0.62	80.09
TB	Amphipods	*Eogammarus confervicolus*	3.94	0.32	1.80	2.49	89.84	2.37	0.75	65.63
CC	Amphipods	*Eogammarus confervicolus*	2.59	1.30	1.54	2.94	118.40	4.27	5.51	52.68
PB	Amphipods	*Eogammarus confervicolus*	4.06	0.39	1.34	1.38	87.68	1.00	0.75	78.42
IP	Amphipods	*Eogammarus confervicolus*	4.53	0.37	1.88	2.45	99.22	0.72	2.34	41.55
IP	Amphipods	*Americorophium salmonis*	9.99	0.51	3.57	8.16	75.63	4.79	3.17	73.33
CC	Amphipods	*Americorophium salmonis*	8.39	0.65	2.96	7.56	111.65	5.21	5.04	202.87
PB	Amphipods	*Americorophium salmonis*	5.78	0.64	1.62	5.07	126.15	1.57	4.43	87.00
CC	Lugworms	*Abarenicola pacifica*	62.63	0.34	12.99	16.68	28.24	26.21	3.93	88.45
PB	Lugworms	*Abarenicola pacifica*	23.87	0.32	11.63	23.57	26.77	19.32	4.06	105.21
WC	Lugworms	*Abarenicola pacifica*	13.69	0.24	9.09	25.06	28.92	16.98	4.32	75.11
WC	Goniadidae worms	*Glycinde picta*	13.35	0.99	1.04	0.91	9.08	2.98	0.27	202.17
WC	Light edged ribbon worm	*Cerebratulus californiensis*	7.82	2.56	0.34	0.31	39.14	0.07	0.16	101.17
CC	Purple Backed Ribbon worms	*Paranemertes peregrina*	25.36	1.13	3.81	0.78	23.18	1.51	5.64	201.74
WC	Purple Backed Ribbon worms	*Paranemertes peregrina*	8.94	1.15	3.50	10.06	23.23	6.15	2.77	134.76
WC	Catworm	*Nephtys caeca*	22.62	4.08	2.30	2.27	10.30	3.64	29.61	142.64
CC	Catworm	*Nephtys caeca*	12.59	6.57	2.94	1.63	30.95	3.25	40.96	180.33
CC	Clam worm	*Nereis vexillosa*	11.22	5.34	4.09	2.32	23.09	2.21	38.28	341.61
WC	Paraonidae worms	*Aricidea hartleyi*	8.31	1.08	14.36	52.78	47.39	21.76	10.71	106.83
CC	Spionidae tube worms	*Streblospio benedicti*	22.57	6.21	6.02	15.91	24.65	10.37	7.00	185.84
WC	Sandworm	*Alitta brandti*	11.71	1.02	4.46	15.13	23.31	6.62	10.74	126.03
